# Factors Associated With Clinician Recommendations for Colorectal Cancer Screening Among Average-Risk Patients: Data From a National Survey

**DOI:** 10.5888/pcd19.210315

**Published:** 2022-04-14

**Authors:** Xuan Zhu, Emily Weiser, Debra J. Jacobson, Joan M. Griffin, Paul J. Limburg, Lila J. Finney Rutten

**Affiliations:** 1 Robert D. and Patricia E. Kern Center for the Science of Health Care Delivery, Mayo Clinic, Rochester, Minnesota; 2 Exact Sciences Corporation, Madison, Wisconsin; 3 Division of Clinical Trials and Biostatistics, Mayo Clinic, Rochester, Minnesota; 4 Division of Health Care Delivery Research, Mayo Clinic, Rochester, Minnesota; 5 Division of Gastroenterology and Hepatology, Mayo Clinic, Rochester, Minnesota; 6 Division of Epidemiology, Mayo Clinic, Rochester, Minnesota

## Abstract

**Introduction:**

Colorectal cancer (CRC) screening among average-risk patients is underused in the US. Clinician recommendation is strongly associated with CRC screening completion. To inform interventions that improve CRC screening uptake among average-risk patients, we examined clinicians’ routine recommendations of 7 guideline-recommended screening methods and factors associated with these recommendations.

**Methods:**

We conducted an online survey in November and December 2019 among a sample of primary care clinicians (PCCs) and gastroenterologists (GIs) from a panel of US clinicians. Clinicians reported whether they routinely recommend each screening method, screening method intervals, and patient age at which they stop recommending screening. We also measured the influence of various factors on screening recommendations.

**Results:**

Nearly all 814 PCCs (99%) and all 159 GIs (100%) reported that they routinely recommend colonoscopy for average-risk patients, followed by stool-based tests (more than two-thirds of PCCs and GIs). Recommendation of other visualization-based methods was less frequent (PCCs, 26%–35%; GIs, 30%–41%). A sizable proportion of clinicians reported guideline-discordant screening intervals and age to stop screening. Guidelines and clinical evidence were most frequently reported as very influential to clinician recommendations. Factors associated with routine recommendation of each screening method included clinician-perceived effectiveness of the method, clinician familiarity with the method, Medicare coverage, clinical capacity, and patient adherence.

**Conclusion:**

Clinician education is needed to improve knowledge, familiarity, and experience with guideline-recommended screening methods with the goal of effectively engaging patients in informed decision making for CRC screening.

**Summary**

**What is already known on this topic?**

Colorectal cancer (CRC) screening among average-risk patients is underused in the US. Clinician recommendation is strongly associated with screening completion.

**What is added by this report?**

We examined clinicians’ routine recommendations of 7 guideline-recommended screening methods and factors associated with these recommendations.

**What are the implications for public health practice?**

A sizable portion of clinicians reported guideline-discordant screening intervals and age to stop screening. Recommendation of screening methods varied by clinician-perceived effectiveness of the method, familiarity with the method, Medicare coverage, clinical capacity, and patient adherence. Clinician education is needed to improve knowledge, familiarity, and experiences with guideline-recommended screening methods with the goal of effectively engaging patients in informed decision making for CRC screening.

**MEDSCAPE CME**

In support of improving patient care, this activity has been planned and implemented by Medscape, LLC and *Preventing Chronic Disease*. Medscape, LLC is jointly accredited by the Accreditation Council for Continuing Medical Education (ACCME), the Accreditation Council for Pharmacy Education (ACPE), and the American Nurses Credentialing Center (ANCC), to provide continuing education for the healthcare team.

Medscape, LLC designates this Journal-based CME activity for a maximum of 1.00 AMA PRA Category 1 Credit(s)™. Physicians should claim only the credit commensurate with the extent of their participation in the activity.

Successful completion of this CME activity, which includes participation in the evaluation component, enables the participant to earn up to 1.0 MOC points in the American Board of Internal Medicine’s (ABIM) Maintenance of Certification (MOC) program. Participants will earn MOC points equivalent to the amount of CME credits claimed for the activity. It is the CME activity provider’s responsibility to submit participant completion information to ACCME for the purpose of granting ABIM MOC credit.

Release date: April 14, 2022; Expiration date: April 14, 2023

**Learning Objectives**

Upon completion of this activity, participants will be able to:

1. Distinguish guidelines for colorectal cancer screening in the United States
2. Identify the preferred method for colorectal cancer screening among gastroenterologists and primary care clinicians
3. Analyze differences in practice patterns between gastroenterologists and primary care clinicians in colorectal cancer screening
4. Assess variables that affect clinicians' choices for colorectal cancer screening modality

EDITOR

Ellen Taratus

Senior Editor

Preventing Chronic Disease 

Disclosure: Ellen Taratus has disclosed no relevant financial relationships.

CME AUTHOR

Charles P. Vega, MD

Health Sciences Clinical Professor of Family Medicine

University of California, Irvine School of Medicine

Irvine, California

Disclosure: Charles P. Vega, MD, has disclosed the following relevant financial relationships:

Served as an advisor or consultant for: GlaxoSmithKline; Johnson & Johnson

AUTHORS

Xuan Zhu, PhD

Mayo Clinic Robert D. and Patricia E. Kern Center for the Science of Health Care Delivery, Rochester, Minnesota

Emily Weiser, MPH

Exact Sciences Corporation, Madison, Wisconsin

Debra J. Jacobson, MS

Division of Clinical Trials and Biostatistics, Mayo Clinic, Rochester, Minnesota

Joan M. Griffin, PhD

Division of Health Care Delivery Research, Mayo Clinic, Rochester, Minnesota

Paul J. Limburg, MD, MPH

Division of Gastroenterology and Hepatology, Mayo Clinic, Rochester, Minnesota

Lila J. Finney Rutten, PhD, MPH

Division of Epidemiology, Mayo Clinic, Rochester, Minnesota

## Introduction

Colorectal cancer (CRC) is the second leading cause of cancer-related death in the US among women and men combined (1,2). Regular screening among asymptomatic populations at average risk of CRC reduces CRC mortality (3,4). Major guideline organizations recommend CRC screening among adults aged 45 to 75 years at average risk of CRC (5,6). Recommended screening options include stool-based tests such as the fecal immunochemical test/guaiac-based fecal occult blood test (FIT/gFOBT) every year and multitarget stool DNA (mt-sDNA) test every 1 to 3 years, and visualization-based methods such as screening colonoscopy every 10 years and computed tomography (CT) colonography and flexible sigmoidoscopy every 5 years (5,6). The Affordable Care Act expanded access to health insurance options for people who were previously uninsured and requires both Medicare and nongrandfathered private health insurance plans (with plan years beginning on or after September 23, 2010) to provide coverage without patient cost sharing for preventive services with a grade of “A” or “B” in US Preventive Services Task Force (USPSTF) recommendations. Currently, CRC screening among average-risk populations has a USPSTF grade of “A” for adults aged 50 to 75 years and a grade “B” for adults aged 45 to 49 years. Nevertheless, despite the availability of multiple effective screening methods and expanded access to health insurance, CRC screening among asymptomatic, average-risk patients continues to be underused in the US (7,8).

Clinician recommendation is consistently reported to play a crucial role in CRC screening completion among average-risk US populations (9,10). In contrast to countries that provide organized national CRC screening programs to average-risk populations, CRC screening in the US occurs on a largely opportunistic, nonprogrammatic basis, where patients either self-refer for screening or receive a recommendation for screening from a clinician during an unrelated health care visit (11,12). CRC screening preferences appear to be misaligned between clinicians and patients: whereas clinicians tend to prefer screening colonoscopy over stool-based tests (13–16), when given options, patients tend to prefer stool-based tests over screening colonoscopy (17,18). Although research is extensive on patient, clinician, and health care system factors associated with CRC screening completion, data on clinician recommendation of guideline-endorsed CRC screening methods and factors associated with guideline-consistent screening recommendations are sparse (19,20). A 2007 survey of a nationally representative sample of primary care clinicians (PCCs) showed that less than 20% made guideline-consistent recommendations across all CRC screening methods (19). A 2018 survey of PCCs in 4 health care systems in the southern and western regions of the US found that while 83% of clinicians rated colonoscopy as very effective for screening eligible patients, only 60% rated FIT as very effective; however, beliefs about test effectiveness were not associated with the likelihood of recommending colonoscopy every 10 years or FIT annually (20). As stool-based tests with improved efficacy are being developed and included in major CRC screening guidelines (5,6), an updated understanding of average-risk CRC screening practices is needed to inform interventions to improve clinician recommendations and screening completion and adherence among patients. The objectives of this study were to 1) characterize clinicians’ routine CRC screening recommendations of guideline-endorsed screening methods among average-risk patients, 2) examine how myriad factors (eg, scientific evidence, clinical practice guidelines) may be associated with these recommendations, and 3) identify barriers to recommending each CRC screening method among clinicians who do not routinely recommend these options to average-risk patients.

## Methods

Our study population was practicing PCCs (defined as clinicians who self-identified as board certified in internal medicine or family medicine) and practicing gastroenterologists (GIs) in the US. Data were collected via a web survey developed by the authors and implemented in November and December 2019 by the National Opinion Research Center at the University of Chicago (http://www.norc.org) using a third-party vendor, Dynata, which maintains a nonprobability panel of more than 200,000 physicians, nurses, and other US health care providers. The health care provider panel was built by recruiting from verified lists (eg, American Hospital Association, American Medical Association) and invitations containing personal identification codes and invitation codes linked to respondents. Respondents were validated at the time of enrollment. Information on the registration form was validated against American Medical Association and National Provider Identifier databases; details such as specialty, medical school, and year of graduation were confirmed. This study was exempted from review by the National Opinion Research Center institutional review board.

We aimed to obtain completed surveys from 750 practicing PCCs and 150 GIs; we aimed for more PCCs than GIs because the panel had more PCCs than GIs. Prior studies that used this panel obtained an average response rate of 10.5% (estimated by Dynata). To reach our target number of 900 respondents, we planned to send the survey via email to 8,600 clinicians. We sent up to 2 reminders to nonresponders within a 21-day period. All participants received remuneration, based on a fair market value hourly rate, for completing the survey. PCCs received the equivalent of $39 and GIs received the equivalent of $51.

### Measures

Questions and response options were adapted from the National Cancer Institute’s National Survey of Colorectal Cancer Screening Practices (21). Clinicians reported whether they routinely recommend to patients each of 7 guideline-endorsed CRC screening methods (colonoscopy, CT colonography, FIT, flexible sigmoidoscopy, flexible sigmoidoscopy with FIT, gFOBT, and mt-sDNA), along with their recommended screening interval for each method and the patient age at which they no longer recommend CRC screening. We referred to the mt-sDNA test as Cologuard because it is the only mt-sDNA test approved by the US Food and Drug Administration for clinical application. We also used 5-point Likert-type scales to measure the level of influence of various factors on screening recommendations in general and clinicians’ routine recommendations of each screening method. For influence on screening recommendations in general, we measured the most up-to-date guidelines of professional societies at the time we fielded the study (the American Cancer Society [5], the US Preventive Services Task Force [22], the American College of Gastroenterology [23], and the US Multi-Society Task Force on Colorectal Cancer [24]); clinical evidence; ease of use; peer support; patient satisfaction; patient adherence; patient preference; and health insurance coverage. For influence on clinicians’ routine recommendations of each screening method, we measured method sensitivity, specificity, familiarity with method, perceived effectiveness, preference for visual inspection, and practice capacity. Additional factors included providers’ perceptions of patient preference, patient adherence, patient satisfaction, and health insurance coverage. The survey presented response options for all questions in randomized order.

### Analysis

Exclusion criteria were not being a PCC or GI and not recommending CRC screening to average-risk patients. We summarized the frequency of responses and used χ^2^ or Fisher exact tests to examine differences by clinical specialty. *P* values were adjusted for multiple testing by using the Benjamini–Hochberg procedure. We chose to control false-discovery rate by using the Benjamini–Hochberg procedure because it gives more power than controlling family-wise error rate (eg, Bonferroni correction) (25). To evaluate the influence of other factors on screening recommendation in general, we assessed the frequency of responses in which clinicians endorsed a factor as “very influential” and used χ^2^ tests to examine differences by clinical specialty. We used multivariable logistic regression to examine associations between the various factors and clinician’s routine recommendations of each screening method, adjusting for provider characteristics including age, sex, race and ethnicity, years of practice, number of patients, number of clinicians in the practice, and self-reported practice location (urban, suburban, rural). We calculated odds ratios (ORs) and 95% CIs with a significant *P* value <.05 after adjusting for multiple testing using the Benjamini–Hochberg procedure (25). We conducted all analyses in R version 3.6.2 by using package *stats* (R Foundation for Statistical Computing).

## Results

**Figure 1:**
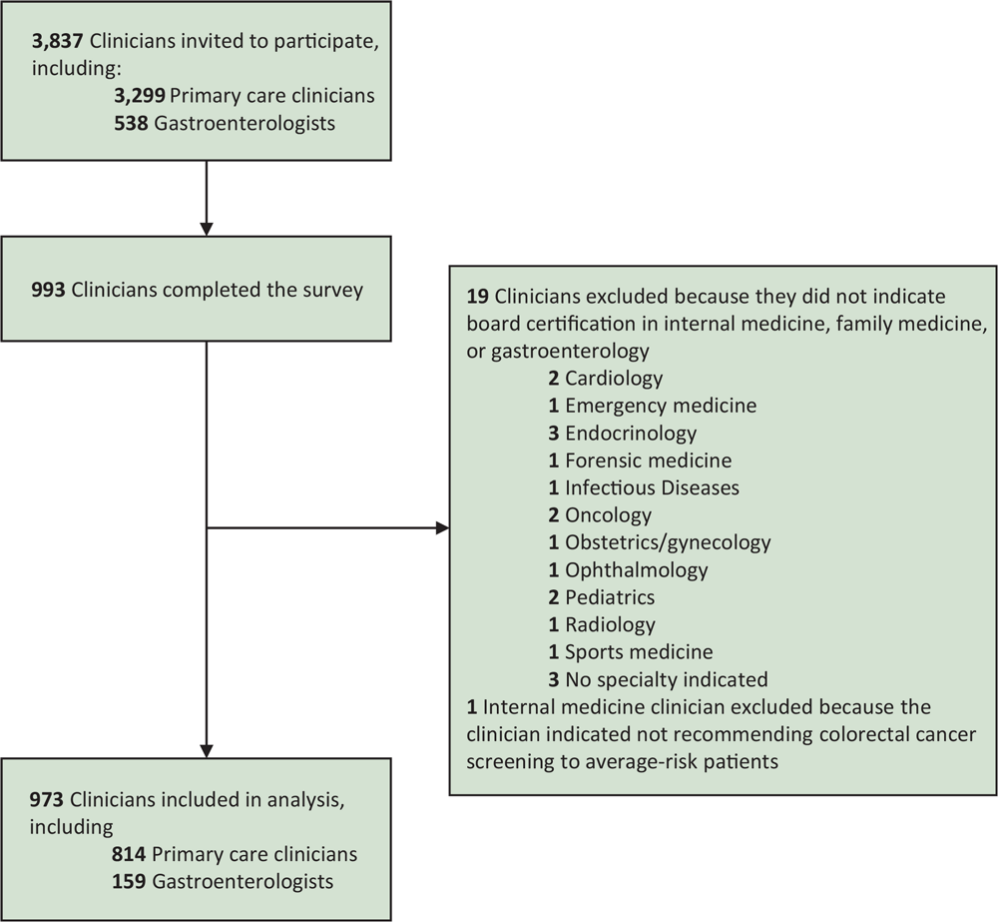
Selection of participants in survey on factors associated with clinician recommendations for colorectal cancer screening among average-risk patients, November–December 2019.

Of the 3,837 surveys sent, 993 clinicians completed the survey, 428 indicated board certification in internal medicine, 387 in family medicine, and 159 in gastroenterology. We excluded clinicians who indicated other specialties (n = 19) and 1 PCC who indicated not recommending CRC screening to average-risk patients, resulting in a final sample of 814 PCCs and 159 GIs (completion rates: PCCs, 24.7%; GIs, 29.6%) (Figure 1). Because response rates were higher than expected, we sent out fewer surveys than planned.

Of the 814 PCCs and 159 GIs, respectively, 44.3% and 52.2% were aged 27 to 49 years, 72.2% and 82.9% were male, 65.6% and 55.4% were non-Hispanic White, and 57.2% and 74.2% had a household income of $200,000 or more (Table 1). Approximately half of PCCs and 40.3% of GIs reported practicing medicine for 20 or more years, and 44.2% and 43.4% reported seeing more than 20 patients on a typical day.

**Table 1:** Clinician and Practice Characteristics of Participants, by Clinical Specialty, in a Survey on Factors Associated With Clinician Recommendations for Colorectal Cancer Screening Among Average-Risk Patients, United States, November–December 2019^a^

Characteristic	Primary care clinicians^b^ (n = 814)	Gastroenterologists (n = 159)
**Age, y^c^**		
27–39	107 (13.1)	41 (25.8)
40–49	254 (31.2)	42 (26.4)
50–59	236 (29.0)	45 (28.3)
≥60	217 (26.7)	31 (19.5)
**Sex^d^**		
Male	586 (72.2)	131 (82.9)
Female	226 (27.8)	27 (17.1)
**Race and ethnicity^e^**		
Hispanic	26 (3.2)	10 (6.3)
Non-Hispanic Asian/Pacific Islander	193 (23.7)	42 (26.4)
Non-Hispanic Black	19 (2.3)	4 (2.5)
Non-Hispanic other^f^/multiple race	42 (5.2)	15 (9.4)
Non-Hispanic White	534 (65.6)	88 (55.4)
**Annual household income, $**		
<74,999	43 (5.3)	4 (2.5)
75,000–124,999	104 (12.8)	9 (5.7)
125,000–174,999	115 (14.1)	12 (7.6)
175,000–199,999	86 (10.6)	16 (10.1)
≥200,000	466 (57.2)	118 (74.2)
**Board certification**		
Internal medicine	427 (52.5)	0
Family medicine	387 (47.5)	0
Gastroenterology	0	159 (100.0)
**No. of years practicing medicine after residency**		
0–9	116 (14.3)	42 (26.4)
10–19	277 (34.0)	53 (33.3)
20–29	271 (33.3)	45 (28.3)
≥30	150 (18.4)	19 (12.0)
**Average no. of patients seen on typical day**		
0–15	163 (20.0)	41 (25.8)
16–20	291 (35.7)	49 (30.8)
21–25	188 (23.1)	30 (18.9)
>25	172 (21.1)	39 (24.5)
**No. of clinicians in practice**		
1–5	344 (42.3)	49 (30.8)
6–15	247 (30.3)	54 (34.0)
≥16	223 (27.4)	56 (35.2)
**Clinician-reported characterization of practice location**		
Urban	262 (32.2)	81 (50.9)
Suburban	447 (54.9)	69 (43.4)
Rural	105 (12.9)	9 (5.7)

a All values presented are number (percentage). The study population included practicing primary care clinicians (PCCs) and practicing gastroenterologists (GIs) in the US in 2019. Information about other clinician or practice characteristics of the study population were not publicly available at the time of the study.

b Includes internal medicine and family medicine practitioners.

c In 2019, 53.6% of PCCs and 50.5% of GIs in the US were aged <55 years (26).

d In 2019, 60% of PCCs and 81.1% of GIs were male (26). Data on sex were missing for 2 primary care clinicians and 1 gastroenterologist.

e In 2018, 50.8% of PCCs and 49.8% of GIs were non-Hispanic White, 18.4% of PCCs and 23.5% of GIs were Asian, 6.2% of PCCs and 5.6% of GIs were Hispanic (alone or with any race), 6.0% of PCCs and 3.7% of GIs were Black or African American, 0.4% of PCCs and 0.1% of GIs were American Indian/Alaska Native, 0.1% of PCCs and 0.1% of GIs were Native Hawaiian/Other Pacific Islander, 0.8% of PCCs and 1% of GIs were non-Hispanic multirace, 0.9% of PCCs and 0.8% of GIs were “other” race or ethnicity, and the race and ethnicity of 16.4% of PCCs and 15.5% of GIs were unknown (27).

f Any race not listed above.

### Routine CRC screening recommendations

Almost all clinicians (99% of PCCs, 100% of GIs) routinely recommended colonoscopy for CRC screening to average-risk patients (Table 2), followed by gFOBT, FIT, and mt-sDNA (85.1%, 79.9%, and 77.1% of PCCs; 75.5%, 78.0%, and 78.0% of GIs, respectively). Routine recommendations of CT colonography, flexible sigmoidoscopy, and flexible sigmoidoscopy with annual FIT were less frequently reported. PCCs more frequently than GIs recommended gFOBT (85% vs 76%, *P* = .009), whereas GIs more frequently than PCCs recommended CT colonography (41% vs 26%, *P* = .001).

**Table 2:** Clinicians’ Recommended Screening Interval and Age to Stop Screening for CRC, by Clinical Specialty, Among Clinicians Who Routinely Recommend These Methods to Asymptomatic, Average-Risk Patients, United States, November–December 2019^a^

Item	Screening method													
	gFOBT		FIT		mt-sDNA (Cologuard)		Colonoscopy		CT colonography		Flexible sigmoidoscopy		Flexible sigmoidoscopy with FIT	
	PCC	GI	PCC	GI	PCC	GI	PCC	GI	PCC	GI	PCC	GI	PCC	GI
No. of clinicians who routinely recommend the method	693 (85.1)	120 (75.5)	650 (79.9)	124 (78.0)	628 (77.1)	124 (78.0)	805 (98.9)	159 (100.0)	213 (26.2)	65 (40.9)	282 (34.6)	48 (30.2)	225 (27.6)	47 (29.6)
*P* value^c^	.009		.73		.82		.43		.001		.49		.73	
Recommended screening interval is consistent with guideline^b^	487 (70.3)	86 (71.7)	370 (56.9)	72 (58.1)	472 (75.2)	96 (77.4)	605 (75.2)	135 (84.9)	109 (51.2)	46 (70.8)	222 (78.7)	31 (64.6)	171 (76.0)	28 (59.6)
*P* value^c^	.81		.81		.81		.03		.03		.06		.049	
**Age to stop screening, y^d^**														
<75	23 (3.3)	2 (1.7)	21 (3.2)	2 (1.6)	25 (4.0)	0	40 (5.0)	2 (1.3)	9 (4.2)	0	18 (6.4)	1 (2.1)	9 (4.0)	1 (2.1)
75	178 (25.7)	42 (35.0)	165 (25.4)	45 (36.3)	159 (25.3)	45 (36.3)	266 (33.0)	56 (35.2)	46 (21.6)	28 (43.1)	75 (26.6)	17 (35.4)	55 (24.4)	19 (40.4)
76–85	200 (28.9)	42 (35.0)	184 (28.3)	43 (34.7)	177 (28.2)	47 (37.9)	258 (32.0)	73 (45.9)	54 (25.4)	19 (29.2)	58 (20.6)	12 (25.0)	52 (23.1)	12 (25.5)
>85	18 (2.6)	3 (2.5)	11 (1.7)	1 (0.8)	13 (2.1)	1 (0.8)	18 (2.2)	2 (1.3)	4 (1.9)	1 (1.5)	9 (3.2)	0	5 (2.2)	0
No upper age limit	274 (39.5)	31 (25.8)	269 (41.4)	33 (26.6)	254 (40.4)	31 (25.0)	223 (27.7)	26 (16.4)	100 (46.9)	17 (26.2)	122 (43.3)	18 (37.5)	104 (46.2)	15 (31.9)
*P* value^c^	.03		.01		.001		.001		<.001		.001		.01	

Abbreviations: CRC, colorectal cancer; CT, computed tomography; FIT, fecal immunochemical test; FOBT, fecal occult blood test; gFOBT, guaiac FOBT; GI, gastroenterologist; mt-sDNA, multitarget stool DNA; PCC, primary care clinician.

a Clinicians were surveyed on factors associated with clinician recommendations for CRC screening among patients at average risk of CRC, November–December 2019. PCCs (n = 814) include internal medicine and family medicine practitioners; 159 GIs participated in survey. All values presented are number (percentage) unless otherwise indicated.

b Recommended screening interval was measured with the following question: “Please share the recommendations you typically make for CRC screening to asymptomatic, average-risk patients for each of the items presented below. Recommended frequency of testing, in years (fill-in-the-blank response). Answers coded as consistent with 2018 American Cancer Society, 2017 Multi-Society Task Force, 2016 US Preventive Services Task Force, or 2009 American College of Gastroenterology CRC screening guidelines if answered gFOBT/FIT every year, mt-sDNA every 1 to 3 years, colonoscopy every 10 years, CT colonography every 5 years, flexible sigmoidoscopy every 5 to 10 years, or flexible sigmoidoscopy every 5 to 10 years with annual FIT.

c *P* values obtained from χ^2^ test or Fisher exact test and adjusted for multiple testing by using the Benjamini–Hochberg procedure; *P* < .05 considered significant.

d Age at which the clinician no longer recommends screening was measured with the following question: “Is there an age at which you no longer recommend screening? If yes, what age?”

Among clinicians who routinely recommended each method, we found differences in their recommended screening intervals and the patient age at which they no longer recommend screening (Table 2). More than 70% of clinicians reported guideline-concordant screening intervals for gFOBT, mt-sDNA, and colonoscopy, while reports of guideline-concordant screening intervals for FIT and CT colonography were less frequent. GIs more frequently than PCCs reported guideline-concordant screening intervals for colonoscopy (84.9% vs 75.2%, *P* = .03) and CT colonography (70.8% vs 51.2%, *P* = .03), while PCCs more frequently reported guideline-concordant screening intervals for flexible sigmoidoscopy with FIT (76.0% vs 59.6%, *P* = .049). For each CRC screening method, GIs more frequently than PCCs reported that they stop recommending screening at age 75, while PCCs more frequently than GIs reported not having an upper age at which they would no longer recommend CRC screening. All differences were significant at *P* < .05.

### Barriers to recommending each CRC screening method

Among 121 PCCs and 39 GIs who reported not routinely recommending gFOBT, inadequate sensitivity (PCCs, 65.3%; GIs, 79.5%) and inadequate specificity (PCCs, 62.0%; GIs, 64.1%) were the most frequently mentioned barriers (Table 3). Among 164 PCCs and 35 GIs who reported not routinely recommending FIT, inadequate sensitivity (PCCs, 33.5%; GIs, 51.4%) and preference for visual inspection (PCCs, 35.4%; GIs, 48.6%) were the most frequently mentioned barriers. PCCs more frequently than GIs reported lack of experience with FIT (32.9% of PCCs vs 2.9% of GIs; *P* = .002). Among 186 PCCs and 35 GIs who reported not routinely recommending mt-sDNA, poor insurance coverage (PCCs, 40.3%; GIs, 34.3%) and preference for visual inspection (PCCs 28.5%; GIs, 48.6%) were the most frequently mentioned barriers. GIs more frequently reported inadequate sensitivity (15.1% of PCCs vs 37.1% of GIs; *P* = .006) and inadequate specificity (13.4% of PCCs vs 42.9% of GIs, *P* < .001) as barriers, while PCCs more frequently reported lack of experience with this method as a barrier (39.2% of PCCs vs 14.3% of GIs, *P* = .009).

**Table 3:** Clinician-Reported Barriers to Recommending Each CRC Screening Method Among Clinicians Who Do Not Routinely Recommend These Methods to Asymptomatic, Average-Risk Patients, United States, November–December, 2019^a^

Item	Screening method												
	gFOBT		FIT		Mt-sDNA (Cologuard)		Colonoscopy^b^	CT colonography		Flexible sigmoidoscopy		Flexible sigmoidoscopy with FIT	
	PCC	GI	PCC	GI	PCC	GI	PCC	PCC	GI	PCC	GI	PCC	GI
No. of clinicians who do not routinely recommend the method	121	39	164	35	186	35	9	601	94	532	111	589	112
**Barrier^c^**													
Inadequate sensitivity (too many false negatives)	79 (65.3)	31 (79.5)	55 (33.5)	18 (51.4)	28 (15.1)	13 (37.1)	1 (11.1)	101 (16.8)	26 (27.7)	230 (43.2)	56 (50.5)	122 (20.7)	42 (37.5)
*P* value^d^	.38		.09		.006		—e	.02^f^		.20		<.001	
Inadequate specificity (too many false positives)	75 (62.0)	25 (64.1)	49 (29.9)	13 (37.1)	25 (13.4)	15 (42.9)	1 (11.1)	85 (14.1)	24 (25.5)	52 (9.8)	13 (11.7)	47 (8.0)	15 (13.4)
*P* value^d^	.81		.48		<.001		—e	.01^f^		.54		.06	
Poor insurance coverage	5 (4.1)	1 (2.6)	30 (18.3)	1 (2.9)	75 (40.3)	12 (34.3)	1 (11.1)	297 (49.4)	44 (46.8)	74 (13.9)	5 (4.5)	98 (16.6)	6 (5.4)
*P* value^d^	—		.07		.60		—e	.64		.009		.003	
Poor patient adherence	18 (14.9)	7 (17.9)	30 (18.3)	7 (20.0)	24 (12.9)	4 (11.4)	4 (44.4)	134 (22.3)	18 (19.1)	193 (36.3)	23 (20.7)	210 (35.7)	26 (23.2)
*P* value^d^	.81		.81		.80		—e	.59		.004		.01^f^	
Preference for visual inspection	52 (43.0)	21 (53.8)	58 (35.4)	17 (48.6)	53 (28.5)	17 (48.6)	2 (22.2)	112 (18.6)	29 (30.9)	67 (12.6)	29 (26.1)	67 (11.4)	32 (28.6)
*P* value^d^	.47		.22		.03		—e	.01^f^		.002		<.001	
Lack of experience with this method	3 (2.5)	0	54 (32.9)	1 (2.9)	73 (39.2)	5 (14.3)	1 (11.1)	231 (38.4)	13 (13.8)	69 (13.0)	3 (2.7)	162 (27.5)	10 (8.9)
*P* value^d^	—		.002		.009		—e	<.001		.004		<.001	

Abbreviations: CRC, colorectal cancer; CT, computed tomography; FIT, fecal immunochemical test; FOBT, fecal occult blood test; gFOBT, guaiac FOBT; GI, gastroenterologist; mt-sDNA, multitarget stool DNA; PCC, primary care clinician.

a Clinicians were surveyed on factors associated with clinician recommendations for colorectal cancer screening among patients at average risk of CRC, November–December 2019. PCCs were internal medicine and family medicine practitioners. All values presented are number (percentage) unless otherwise indicated.

b All GIs reported routinely recommending colonoscopy for CRC screening.

c Barrier to each method was measured with the following question: “For each of the following CRC screening options, please identify any factors that prevent you from recommending that method to asymptomatic, average-risk patients age 50 and older. Please select all that apply.”

d *P* values obtained from χ^2^ test or Fisher exact test and adjusted for multiple testing by using the Benjamini–Hochberg procedure.

e Analysis not conducted because outcome was rare.

f Statistical test did not have 80% power to detect this difference.

Among the 9 PCCs who reported not routinely recommending colonoscopy, the most frequently reported barrier was poor patient adherence (44.4%). Among 601 PCCs and 94 GIs who reported not routinely recommending CT colonography, poor insurance coverage (PCCs, 49.4%; GIs, 46.8%) was the most frequently mentioned barrier. PCCs more frequently reported lack of experience as a barrier to CT colonography (38.4% of PCCs vs 13.8% of GIs, *P* < .001). Among 532 PCCs and 111 GIs who reported not routinely recommending flexible sigmoidoscopy, inadequate sensitivity (PCCs, 43.2%; GIs, 50.5%) was the most frequently mentioned barrier, followed by poor patient adherence (PCCs, 36.3%; GIs, 20.7%). GIs more frequently reported preference for visual inspection (12.6% of PCCs vs 26.1% of GIs, *P* = .002). Among 589 PCCs and 112 GIs who reported not routinely recommending flexible sigmoidoscopy with annual FIT, PCCs more frequently reported poor patient adherence (35.7% of PCCs vs 23.2% of GIs, *P* = .01) and lack of experience with this method (27.5% of PCCs vs 8.9% of GIs, *P* < .001), while GIs more frequently reported inadequate sensitivity (20.7% of PCCs vs 37.5% of GIs, *P* < .001) and preference for visual inspection (11.4% of PCCs vs 28.6% of GIs, *P* < .001) as barriers.

### Factors associated with CRC screening recommendations

About 60% of PCCs and around half of GIs reported American Cancer Society 2018 and USPSTF 2016 guidelines as being very influential to their CRC screening recommendations (Table 4). GIs more frequently than PCCs reported American College of Gastroenterology 2009 and Multi-Society Task Force 2017 guidelines as being very influential (72.3% vs 46.1%, *P* < .001; 50.3% vs 35.9%, *P* = .005). Factors that were most frequently reported as very influential included published clinical evidence (69.5% of PCCs and 78.5% of GIs) and inclusion in clinical practice guidelines (53.0% of PCCs vs 65.2% of GIs, *P* = .02), followed by ease of use within practice (45.0% of PCCs and 51.3% of GIs), patient likelihood to comply with recommendation (47.6% of PCCs and 45.3% of GIs), and patient satisfaction with recommended method (46.9% of PCCs and 41.4% of GIs).

**Table 4:** Influence of Guidelines and Method-Specific Factors on Clinician CRC Screening Recommendation to Asymptomatic, Average-Risk Patients, by Provider Specialty, United States, November–December 2019^a^

Item	Rated as very influential, no. (%)		*P* value^b^
	Primary care clinicians (n = 814)	Gastroenterologists (n = 159)	
**CRC screening clinical practice guidelines^c^**			
American Cancer Society Colorectal Cancer Screening Guideline (5)	451 (57.8)	82 (51.6)	.23
US Preventive Services Task Force Colorectal Cancer Screening Guideline (22)	478 (61.4)	86 (54.4)	.21
American College of Gastroenterology Colorectal Cancer Screening Guideline (23)	349 (46.1)	115 (72.3)	<.001
Multi-Society Task Force Colorectal Cancer Screening Guideline (24)	245 (35.9)	77 (50.3)	.005
**Method-specific factors^d^**			
Published clinical evidence	557 (69.5)	124 (78.5)	.07
Inclusion in clinical practice guidelines	428 (53.0)	103 (65.2)	.02
Ease of use in practice	363 (45.0)	81 (51.3)	.23
Support among peer groups and professional societies/networks	244 (30.3)	62 (39.0)	.08
Patient satisfaction with recommended method	380 (46.9)	65 (41.4)	.27
Patient likelihood to comply with recommendation	383 (47.6)	72 (45.3)	.60
Patient request for specific method	308 (38.0)	53 (33.5)	.34
Patient insurance coverage	303 (37.6)	64 (40.5)	.54

Abbreviation: CRC, colorectal cancer.

a Clinicians were surveyed on factors associated with clinician recommendations for colorectal cancer screening among patients at average risk of CRC, November–December 2019. Primary care clinicians include internal medicine and family medicine practitioners. All values presented are number (percentage) unless otherwise indicated.

b *P* values obtained from χ^2^ test or Fisher exact test and adjusted for multiple testing by using the Benjamini–Hochberg procedure.

c Influence of the guidelines was measured with the following question: “Please rate the following CRC screening clinical practice guidelines based on how much they influence your recommendation of specific CRC screening methods. Please use a scale from 1 to 5, where 1 is not at all influential and 5 is very influential.” Clinicians who reported not knowing the guidelines were excluded (American Cancer Society guidelines, 34 primary care physicians; US Preventive Services Task Force guidelines, 35 primary care physicians and 1 gastroenterologist; American College of Gastroenterology guidelines, 57 primary care physicians; Multi-Society Task Force guidelines, 131 primary care physicians and 6 gastroenterologists).

d Influence of the method-specific factors was measured with the following question: “Please rate the level of influence the following method-specific factors have on your recommendation of specific CRC screening methods. Please use a scale from 1 to 5, where 1 is not at all influential and 5 is very influential.” Not all physicians answered this question; missingness for each question ranged from 4 to 12 among primary care physicians and 0 to 2 among gastroenterologists; denominators for percentages vary.

**Figure 2:**
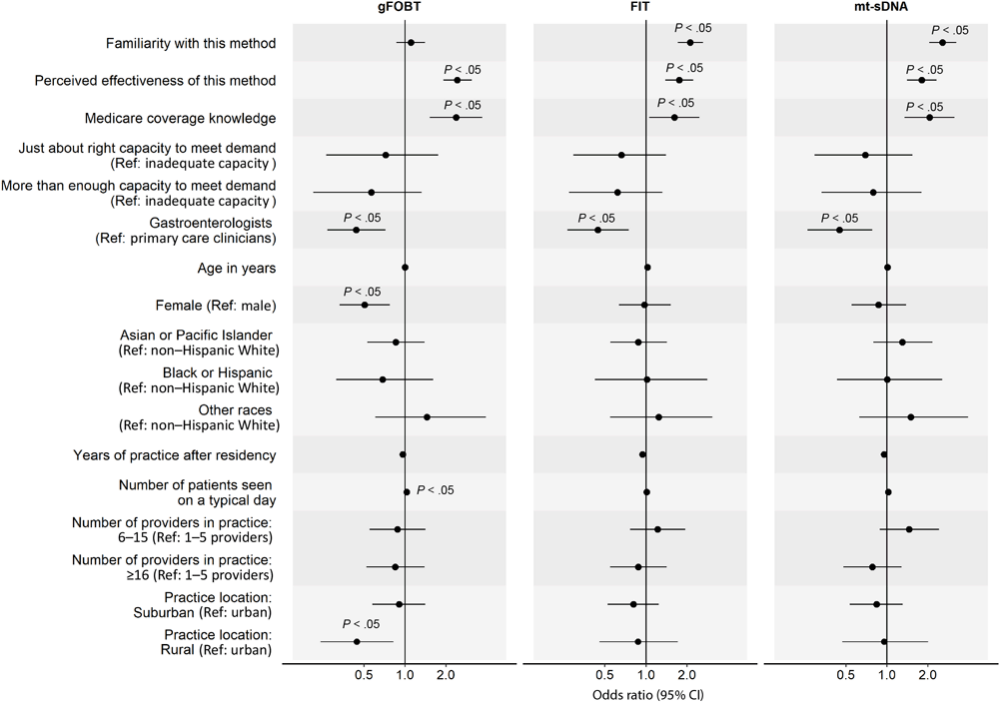
Factors associated with clinicians routinely recommending any of 3 of the stool-based colorectal cancer screening (CRC) methods to average-risk patients. Familiarity was measured with “Please rate your level of familiarity with the following CRC screening methods on a scale from 1 to 5, where 1 is not at all familiar and 5 is very familiar.” Perceived effectiveness was measured with “Please rate how effective the following screening methods are at reducing CRC mortality among patients who are at average risk for CRC and age 50 or older” on a 5-point scale, where 1 is not at all effective and 5 is very effective. Medicare coverage knowledge was measured with “To the best of your knowledge, does Medicare cover the following CRC screening options for asymptomatic, average-risk patients age 50 years and older with no out-of-pocket costs to patients?” Response options were yes, no, and don’t know. We combined data on Black and Hispanic clinicians because of small sample sizes. *P* values were adjusted using Benjamini–Hochberg procedure. Abbreviations: gFOBT, guaiac-based fecal occult blood test; FIT, fecal immunochemical test; mt-sDNA, multitarget stool DNA; ref, reference.

**Figure 3:**
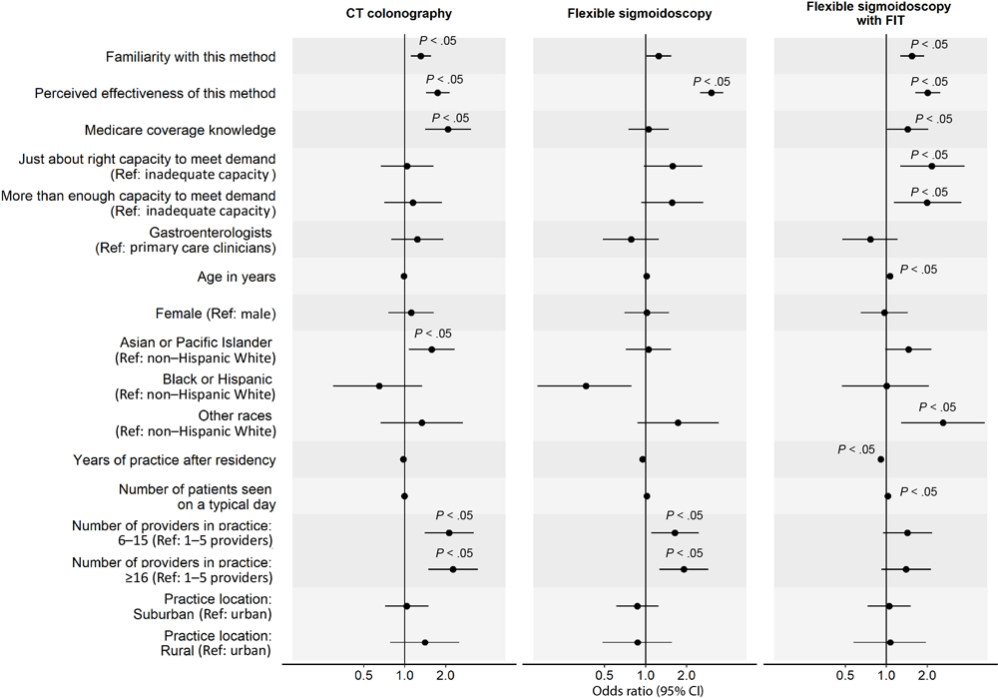
Factors associated with clinicians routinely recommending each visualization-based colorectal cancer screening (CRC) method to average-risk patients. Analysis on colonoscopy was omitted because only 1% of primary care physicians did not recommend colonoscopy for CRC screening; thus, we found no variability in this outcome. Familiarity was measured with “Please rate your level of familiarity with the following CRC screening methods on a scale from 1 to 5, where 1 is not at all familiar and 5 is very familiar.” Perceived effectiveness was measured with “Please rate how effective the following screening methods are at reducing CRC mortality among patients who are at average risk for CRC and age 50 or older” on a 5-point scale, where 1 is not at all effective and 5 is very effective. Medicare coverage knowledge was measured with “To the best of your knowledge, does Medicare cover the following CRC screening options for asymptomatic, average-risk patients age 50 years and older with no out-of-pocket costs to patients?” Response options were yes, no, and don’t know. We combined data on Black and Hispanic clinicians because of small sample sizes. *P* values were adjusted using Benjamini–Hochberg procedure. Abbreviations: CT, computed tomography; FIT, fecal immunochemical test; ref, reference.

In the analysis of factors associated with clinicians’ routine recommendation of each stool-based and visualization-based screening method (Figure 2 and Figure 3), we omitted colonoscopy because only 1% of PCCs did not routinely recommend it. For all screening methods, a higher perceived effectiveness of the method at reducing CRC mortality was associated with a higher likelihood of routine recommendation (ORs range, 1.74–3.05; *P* < .05 for all). Higher familiarity with the screening method was associated with a higher likelihood of routine recommendation for FIT (OR, 2.11; 95% CI, 1.71–2.62), mt-sDNA (OR, 2.55; 95% CI, 2.04–3.23), CT colonography (OR, 1.31; 95% CI, 1.11–1.56), and flexible sigmoidoscopy with annual FIT (OR, 1.54; 95% CI, 1.27–1.89). Clinician’s belief that Medicare covers the method without out-of-pocket costs to patients was associated with a higher likelihood of routine recommendation for gFOBT (OR, 2.37; 95% CI, 1.52–3.69), mt-sDNA (OR, 2.05; 95% CI, 1.35–3.14), and CT colonography (OR, 2.08; 95% CI, 1.41–3.06). Having just enough (OR, 2.15; 95% CI, 1.27–3.75) or more than enough (OR, 1.99; 95% CI, 1.14–3.56) practice capacity (vs inadequate capacity) to meet patient demand for the method was associated with a higher likelihood of routine recommendation for flexible sigmoidoscopy with annual FIT.

**Table:** 

Variable	Odd ratio (95% CI)		
	gFOBT	FIT	mt-sDNA
Familiarity with this method	1.1 (0.86–1.4)	2.11 (1.71–2.62)^a^	2.55 (2.04–3.23)^a^
Perceived effectiveness of this method	2.42 (1.92–3.1)^a^	1.75 (1.39–2.22)^a^	1.8 (1.4–2.31)^a^
Medicare coverage knowledge	2.37 (1.52–3.69)^a^	1.62 (1.06–2.47)^a^	2.05 (1.35–3.14)^a^
**Practice capacity**			
Inadequate capacity	1 [Reference]	1 [Reference]	1 [Reference]
Just about right capacity to meet demand	0.72 (0.26–1.75)	0.66 (0.29–1.4)	0.69 (0.29–1.54)
More than enough capacity to meet demand	0.56 (0.21–1.32)	0.62 (0.27–1.32)	0.79 (0.33–1.79)
**Clinical specialty**			
Primary care clinicians	1 [Reference]	1 [Reference]	1 [Reference]
Gastroenterologists	0.44 (0.27–0.72)^a^	0.44 (0.27–0.75)^a^	0.45 (0.26–0.78)^a^
**Age in years**	1.00 (0.96–1.05)	1.03 (0.98–1.07)	1.01 (0.96–1.06)
**Sex**			
Female	0.51 (0.33–0.77)^a^	0.98 (0.63–1.52)	0.87 (0.55–1.38)
Male	1 [Reference]	1 [Reference]	1 [Reference]
**Race or ethnicity**			
Non-Hispanic White	1 [Reference]	1 [Reference]	1 [Reference]
Asian or Pacific Islander	0.85 (0.53–1.4)	0.88 (0.55–1.42)	1.3 (0.8–2.15)
Black or Hispanic	0.68 (0.31–1.61)	1.02 (0.42–2.81)	1 (0.43–2.54)
Other races	1.45 (0.6–3.91)	1.24 (0.55–3.06)	1.5 (0.63–3.93)
**Years of practice after residency**	0.96 (0.91–1.01)	0.94 (0.89–0.99)	0.95 (0.9–1.01)
**Number of patients seen on a typical day**	1.03 (1.01–1.06)^a^	1.01 (0.99–1.04)	1.02 (1–1.05)
**Number of providers in practice**			
1–5	1 [Reference]	1 [Reference]	1 [Reference]
6–15	0.88 (0.55–1.42)	1.22 (0.77–1.95)	1.45 (0.88–2.42)
≥16	0.85 (0.52–1.39)	0.88 (0.55–1.42)	0.78 (0.48–1.28)
**Location of practice**			
Urban	1 [Reference]	1 [Reference]	1 [Reference]
Suburban	0.9 (0.58–1.41)	0.81 (0.52–1.24)	0.84 (0.53–1.31)
Rural	0.44 (0.24–0.82)^a^	0.87 (0.46–1.71)	0.95 (0.47–2.01)

^a^ *P* < .05.

**Table:** 

Variable	Odds ratio (95% CI)		
	CT colonography	Flexible sigmoidoscopy	Flexible sigmoidoscopy with FIT
Familiarity with this method	1.31 (1.11–1.56)^a^	1.25 (1.02–1.54)	1.54 (1.27–1.89)^a^
Perceived effectiveness of this method	1.74 (1.43–2.14)^a^	3.05 (2.51–3.73)^a^	2.01 (1.63–2.49)^a^
Medicare coverage knowledge	2.08 (1.41–3.06)^a^	1.05 (0.75–1.48)	1.43 (1.01–2.02)^a^
**Practice capacity**			
Inadequate capacity	1 [Reference]	1 [Reference]	1 [Reference]
Just about right capacity to meet demand	1.04 (0.67–1.63)	1.58 (0.97–2.61)	2.15 (1.27–3.75)^a^
More than enough capacity to meet demand	1.15 (0.71–1.88)	1.56 (0.93–2.65)	1.99 (1.14–3.56)^a^
**Clinical specialty**			
Primary care clinicians	1 [Reference]	1 [Reference]	1 [Reference]
Gastroenterologists	1.24 (0.80–1.92)	0.78 (0.49–1.25)	0.76 (0.48–1.21)
**Age in years**	0.99 (0.95–1.02)	1.02 (0.98–1.05)	1.06 (1.02–1.1)^a^
**Sex**			
Female	1.12 (0.76–1.64)	1.02 (0.70–1.48)	0.97 (0.65–1.43)
Male	1 [Reference]	1 [Reference]	1 [Reference]
**Race or ethnicity**			
Non-Hispanic White	1 [Reference]	1 [Reference]	1 [Reference]
Asian or Pacific Islander	1.58 (1.07–2.32)^a^	1.05 (0.71–1.54)	1.45 (0.98–2.15)
Black or Hispanic	0.65 (0.30–1.34)	0.37 (0.16–0.79)	1 (0.47–2.05)
Other races	1.34 (0.66–2.67)	1.73 (0.87–3.43)	2.61 (1.29–5.29)^a^
**Years of practice after residency**	0.98 (0.94–1.02)	0.95 (0.91–0.99)	0.91 (0.87–0.95)^a^
**Number of patients seen on a typical day**	0.99 (0.98–1.01)	1.02 (1–1.04)	1.02 (1.01–1.04)^a^
**Number of providers in practice**			
1–5	1 [Reference]	1 [Reference]	1 [Reference]
6–15	2.12 (1.40–3.21)^a^	1.64 (1.10–2.46)^a^	1.43 (0.95–2.16)
≥16	2.26 (1.49–3.45)^a^	1.91 (1.26–2.89)^a^	1.4 (0.92–2.12)
**Location of practice**			
Urban	1 [Reference]	1 [Reference]	1 [Reference]
Suburban	1.03 (0.72–1.49)	0.87 (0.61–1.24)	1.05 (0.73–1.51)
Rural	1.41 (0.78–2.51)	0.87 (0.48–1.56)	1.07 (0.57–1.95)

^a^ *P* < .05.

GIs were less likely than PCCs to report routine recommendation for stool-based tests (gFOBT: OR, 0.44; 95% CI, 0.27–0.72; FIT: OR, 0.44; 95% CI, 0.27–0.75; mt-sDNA: OR, 0.45; 95% CI, 0.26–0.78), while female clinicians were less likely to report routine recommendation for gFOBT (OR, 0.51; 95% CI, 0.33–0.77). Clinicians who identified as other or multirace (versus non-Hispanic White) were more likely to report routine recommendation for flexible sigmoidoscopy with annual FIT (OR, 2.61; 95% CI, 1.29–5.29). More years of practice after residency was associated with a lower likelihood of routine recommendation for flexible sigmoidoscopy with annual FIT (OR, 0.91; 95% CI, 0.87–0.95) and seeing more patients on a typical day was associated with a higher likelihood of routine recommendation for gFOBT (OR, 1.03; 95% CI, 1.01–1.06). Clinicians who were from practices with 6 or more providers (vs 1–5 providers) were more likely to report routine recommendation for CT colonography (6–15 providers: OR, 2.12; 95% CI, 1.40–3.21; ≥16 providers: OR, 2.26; 95% CI, 1.49–3.45) and those from practices with 16 or more providers were more likely to report routine recommendation for flexible sigmoidoscopy (OR, 1.91; 95% CI, 1.26–2.89). Lastly, clinicians who reported that their practice was in a rural (vs urban) location were less likely to report routine recommendation for gFOBT (OR, 0.44; 95% CI, 0.24–0.82).

## Discussion

Our national survey of PCCs and GIs showed that almost all clinicians routinely recommended colonoscopy for CRC screening to average-risk patients, followed by the available stool-based tests (among 75.5% to 85.1% of clinicians). Routine recommendation of other visualization-based screening methods (CT colonography, flexible sigmoidoscopy) was much less frequent (among 26.2% to 40.9% of clinicians). This pattern suggests that among all available guideline-endorsed CRC screening methods, including newer stool-based tests with improved efficacy, screening colonoscopy remains US clinicians’ preferred method for average-risk CRC screening (13–16).

We found differences in clinicians’ recommended screening intervals and the patient age at which they no longer recommend screening; a large proportion of clinicians’ responses were inconsistent with major CRC screening guidelines. Among clinicians who reported routinely recommending each method, reports of guideline-discordant screening intervals were common for FIT and CT colonography among PCCs and common for FIT, flexible sigmoidoscopy, and flexible sigmoidoscopy with FIT among GIs. Although less frequent than other methods, reports of guideline-discordant screening intervals for colonoscopy were still sizable: 24.8% of PCCs and 15.1% of GIs. These findings suggest that potential deficits exist in clinician knowledge about newer tests and less commonly used methods. Clinician education that covers all guideline-recommended screening methods may be needed to improve clinician knowledge, and health system–level interventions to implement CRC screening guidelines may enhance the delivery of guideline-concordant care.

Most clinicians surveyed reported an age other than 75 years as the age at which they no longer recommend CRC screening, and a considerable proportion of clinicians reported that they did not have an upper age limit for CRC screening. Reports of guideline-discordant upper age limits for recommending screening were more frequent among PCCs than GIs. In light of research showing overuse of CRC screening among certain populations (eg, older patients with limited life expectancies [28–30]), these findings suggest that CRC screening guidelines are not adequately followed. The USPSTF 2021 guidelines assigned a grade “C” for CRC screening for adults aged 76 to 85 years and recommend that clinicians selectively offer CRC screening to patients in this age group and consider the patient’s overall health, prior screening history, and preferences when determining whether screening is appropriate (6). Clinicians could focus the explanation on shifting the priority to other health issues and frame the discussion around lack of benefit from CRC screening without necessarily mentioning life expectancy (31,32).

Barriers to recommending stool-based tests were similar to those found in previous research (14,16), with major concerns being inadequate sensitivity and inadequate specificity, followed by preference for visual inspection; concerns about inadequate sensitivity and specificity were more prominent for gFOBT than for FIT and mt-sDNA. Patients may prefer less invasive options; provider recommendations that align with patient preferences may improve CRC screening use (17,18,33). Additionally, mailed outreach of stool-based tests has been shown to be effective at improving CRC screening uptake in communities with poor colonoscopy access and in reducing CRC screening disparities (34–36). Given our findings, education efforts may be needed to provide clinicians with a more accurate and complete picture of CRC screening, including up-to-date clinical evidence on all guideline-recommended methods with consideration of patient, clinician, and health system factors that may impact the effectiveness of each method. These efforts may be especially needed among PCCs, given that nearly 40% of PCCs reported lack of experience with newer stool-based tests. Given that the best screening method is the one that the patient is most likely to complete, clinicians may benefit from training on shared decision-making approaches to better incorporate patient needs, preferences, and values in CRC screening recommendations to maximize screening uptake and adherence. Discussing all 7 guideline-recommended screening options in one sitting with patients may be impractical given the time constraint of the typical patient visit and the risk of information overload for the patient; we suggest clinicians offer patients a choice between multiple screening options based on patient needs, preferences, and values and the availability of the screening modalities in their practice. Additionally, decision aids and clinical conversation aids that help break CRC screening decisions into discrete, manageable steps may be promising approaches to increase shared and informed CRC screening decisions (37–39). When discussing stool-based tests, clinicians should emphasize the importance of completing follow-up colonoscopy if the stool-based test returns a positive result and address potential barriers including access to colonoscopy and out-of-pocket costs. Poor insurance coverage was cited as a barrier to recommending CT colonography by about half of the clinicians, echoing previous research showing out-of-pocket expenses as a major barrier for patients to undergo CT colonography for CRC screening (40,41). Currently, Medicare does not cover the cost of screening CT colonography; many major private health insurance carriers and states have approved mandatory coverage for screening CT colonography (42–44). Concerns about insurance coverage for mt-sDNA were also frequent, despite Medicare and most private insurance plans already covering this test with no out-of-pocket cost for CRC screening–eligible patients (45).

Major CRC screening guidelines, published clinical evidence, and inclusion in clinical practice guidelines were most frequently reported as very influential in clinicians’ decisions on CRC screening recommendations, while patient requests for a specific method, patient insurance coverage, and support among peer groups were less frequently cited. Most GIs cited 2009 American College of Gastroenterology guidelines as very influential to their practice, while most PCCs chose 2018 American Cancer Society and 2016 USPSTF guidelines, which may in part explain the differences in screening recommendations between PCCs and GIs. This difference between PCCs and GIs likely was due to differences in training and clinicians’ greater familiarity with and confidence in their professional organization’s guidelines. Factors associated with clinicians’ routine recommendation of specific screening methods included perceived effectiveness of the method at reducing CRC mortality, familiarity with the method, knowledge about Medicare coverage, and clinical capacity (for flexible sigmoidoscopy only). These findings underscore the need for education at the health system level to improve clinician knowledge of clinical evidence, guideline-concordant usage, and insurance coverage regarding all available CRC screening methods.

We also observed differences in routine recommendation of certain CRC screening methods by clinician and practice characteristics. For example, GIs were less likely to recommend stool-based tests than PCCs. This may in part be because GIs were more likely to follow the American College of Gastroenterology screening guidelines in which colonoscopy was recommended as the preferred strategy for CRC screening. Clinicians from larger practices were more likely to recommend CT colonography and flexible sigmoidoscopy than those from smaller practices with 1 to 5 providers, likely reflecting differences in clinical capacities and access regarding these less commonly used modalities. Clinicians who reported practicing in rural locations were less likely than those in urban locations to recommend gFOBT, and clinicians who identified as non-Hispanic “other” race or multiple races, compared with non-Hispanic White clinicians, were more likely to recommend flexible sigmoidoscopy with FIT. These findings may reflect differences in clinician training, patient populations, and practices’ access to screening methods. It may be beneficial for practices to implement community-level outreach and educational efforts to increase the public’s awareness of available CRC screening methods and each method’s unique attributes, with the goal of facilitating effective patient–provider communication and informed decision making regarding CRC screening.

Our study has several limitations. First, the cross-sectional survey design precludes the evaluation of causal associations. Second, we relied on clinician self-report, which is prone to recall bias, to assess routine CRC screening recommendations. Future research may benefit from using electronic health record data to confirm clinician self-reported data. Third, although we measured the influence of patient health insurance coverage on provider recommendation, we did not measure how health insurance coverage and out-of-pocket cost for follow-up colonoscopy influenced provider recommendation of stool-based tests. Future research may benefit from differentiating the influence of insurance coverage for screening versus follow-up testing on provider recommendation of screening options. Fourth, we combined Black and Hispanic clinicians into 1 category in certain analyses because of their small sample sizes. Future research may benefit from oversampling clinicians from underrepresented population groups in medical professions to ensure diverse experiences and perspectives are captured. Fifth, because of the relatively small sample size of GIs, we were unable to examine interaction effects between clinical specialty and various factors with sufficient statistical power. Sixth, although consistent with declining and generally lower response rates for clinician surveys, our completion rate was limited for both PCCs and GIs, which may have introduced selection bias (46,47). Nonrespondents may have differed from respondents in clinician and practice characteristics, and their routine CRC screening recommendation practices and perceived influence of various factors on screening recommendation decision making may therefore have differed. Finally, the survey sample is not nationally representative of the practicing PCC and GI population, limiting the generalizability of the findings. However, we achieved a relatively diverse sample by using a national panel of US clinicians.

Our national survey of clinicians showed that colonoscopy remains the most recommended CRC screening method, followed by the available stool-based tests. Routine recommendation of CT colonography and flexible sigmoidoscopy were less common. Although clinical evidence and CRC screening guidelines were most frequently cited as very influential in clinicians’ decisions on CRC screening recommendations, we found discrepancies between clinician-reported practices and major CRC screening guidelines, particularly in patient age to stop screening. Our findings suggest a need for clinician training and education to improve knowledge, familiarity, and experiences with all available CRC screening methods, especially newer methods, with the goal of effectively engaging average-risk patients in informed decision making regarding CRC screening.

## Post-Test Information

To obtain credit, you should first read the journal article. After reading the article, you should be able to answer the following, related, multiple-choice questions. To complete the questions (with a minimum 75% passing score) and earn continuing medical education (CME) credit, please go to http://www.medscape.org/journal/pcd. Credit cannot be obtained for tests completed on paper, although you may use the worksheet below to keep a record of your answers.

You must be a registered user on http://www.medscape.org. If you are not registered on http://www.medscape.org, please click on the “Register” link on the right hand side of the website.

Only one answer is correct for each question. Once you successfully answer all post-test questions, you will be able to view and/or print your certificate. For questions regarding this activity, contact the accredited provider, CME@medscape.net. For technical assistance, contact CME@medscape.net. American Medical Association’s Physician’s Recognition Award (AMA PRA) credits are accepted in the US as evidence of participation in CME activities. For further information on this award, please go to https://www.ama-assn.org. The AMA has determined that physicians not licensed in the US who participate in this CME activity are eligible for AMA PRA Category 1 Credits™. Through agreements that the AMA has made with agencies in some countries, AMA PRA credit may be acceptable as evidence of participation in CME activities. If you are not licensed in the US, please complete the questions online, print the AMA PRA CME credit certificate, and present it to your national medical association for review.

## Post-Test Questions

### Study Title: Factors Associated With Clinician Recommendations for Colorectal Cancer Screening Among Average-Risk Patients: Data From a National Survey

#### CME Questions

1. Which one of the following statements regarding US recommendations for screening for colorectal cancer is most accurate?
  - Screening exams should be performed among average-risk adults between the ages of 50 and 80 years
  - The screening interval between normal colonoscopies is every 7 years
  - The screening interval between normal fecal immunochemical tests (FITs) is 1 year
  - The screening interval between normal computed tomography (CT) colonography is every 3 years
2. What was the most preferred method of screening for colorectal cancer (CRC) among primary care clinicians (PCCs) and gastroenterologists (GIs) in the current study?
  - FIT
  - Colonoscopy
  - Multitarget stool DNA
  - Flexible sigmoidoscopy
3. Which one of the following differences in comparing the practice characteristics of GIs and PCCs was noted in the current study?
  - PCCs were more likely to adhere to guideline intervals for screening colonoscopy
  - PCCs were more likely to recommend screening after age 75 years
  - PCCs reported more experience with FIT
  - PCCs were more likely to recommend CT colonography
4. Which one of the following variables was most influential in participants' decision for CRC screening modality in the current study?
  - Patient insurance coverage
  - Patient likelihood of adherence
  - Clinical practice guidelines
  - Published clinical evidence
